# Early Documentation of Life-Sustaining Treatment Orders Prior to Diagnosis of COVID-19 in Nursing Home Residents

**DOI:** 10.1093/geroni/igab046.2698

**Published:** 2021-12-17

**Authors:** David Henkin, Brande Harris, Sara Espinoza, Hazel Caliz, Kimberly Oakman, Sandra Sanchez-Reilly, Marcos Restrepo

**Affiliations:** 1 University of Texas HSC at San Antonio, Detroit , Michigan, United States; 2 South Texas Veterans Health Care System, South Texas Veterans Health Care System, Texas, United States; 3 University of Texas Health Science Center San Antonio, San Antonio, Texas, United States; 4 South Texas Veterans Health Care System, San Antonio, Texas, United States; 5 University of Texas HSC San Antonio, San Antonio, Texas, United States

## Abstract

Coronavirus disease 2019 (COVID-19) has had a devastating impact on older adult nursing home residents (NHR). NHRs comprise greater than one-third of COVID-19 U.S. deaths, emphasizing the importance of engaging in end-of-life discussions. At South Texas Veterans Health Care System (STVHCS), we implemented early documentation of patient’s Life-Sustaining Treatment (LST) or end-of-life goals-of-care preferences prior COVID-19 infection. We now aim to examine the association between early LST documentation (prior to COVID-19 diagnosis) and hospital admissions for COVID-19 by conducting a retrospective cohort study of Veteran NHRs at STVHCS from March 2020-January 2021. Inclusion criteria were NHRs with COVID-19 diagnosis, LST documentation, and clear timing of whether the LST documentation occurred before or after COVID-19 diagnosis. Logistic regression was used to determine the likelihood of hospitalization by whether LST was documented before or after COVID-19 diagnosis. 208 NHRs were diagnosed with COVID-19 and 160 (76.9%) had LST documentation. Of these, 148 were included in the analysis: 84 (56.8%) had a completed LST note prior to diagnosis and 64 (43.2%) after diagnosis. The hospitalization rate was 46% for those with LST prior to diagnosis compared to 78% in those with LST after diagnosis (OR = 0.24, 95% CI: 0.12-0.50, P<0.001), showing that early LST documentation was associated with 76% lower likelihood of hospitalization. Early interventions for LST documentation can reduce hospitalization in high-risk populations. These findings may have implications for reducing unnecessary hospitalizations, diminishing healthcare costs, and resolving ethical dilemmas related to potential resource allocation during a pandemic.

